# "*Mycobacterium tilburgii*" Infections

**DOI:** 10.3201/eid1203.051139

**Published:** 2006-03

**Authors:** Dirk Wagner, Margreet C. Vos, Anton G.M. Buiting, Annerose Serr, Anneke M.C. Bergmans, Winfried V. Kern, Leo M. Schouls

**Affiliations:** * University Hospital, Freiburg, Germany;; † Erasmus University Medical Center, Rotterdam, the Netherlands;; ‡ St Elisabeth Hospital, Tilburg, the Netherlands;; § Franciscus Hospital, Roosendaal, the Netherlands;; ¶ National Institute of Public Health and the Environment, Bilthoven, the Netherlands

**Keywords:** Mycobacterium Infections, Atypical, AIDS, Cystitis, Gastrointestinal Diseases, Polymerase Chain Reaction

**To the Editor:** Advanced molecular biologic methods have improved the species differentiation and taxonomic classification of microorganisms, including nontuberculous mycobacteria. Identifying and characterizing an increasing number of "new" mycobacterial species of medical importance is now possible. Often, these newly described mycobacteria have been isolated from immunocompromised patients (*1**,**2*). Some of those have been difficult to cultivate, and 16S rRNA gene sequencing or similar methods have become of major importance to allow species identification and clinical diagnosis. Here, we report 2 patients with disease likely caused by a novel mycobacterial species that could not been previously cultivated. Diagnosis relied on molecular identification of acid-fast organisms in tissues. We also briefly review 2 similar cases published previously and note that all 4 known patients were from central Europe.

A 43-year-old woman without evidence of immunodeficiency reported recurrent episodes of dysuria, hematuria, and abdominal discomfort for >1 year. On cystoscopy, a hyperemic bladder with yellow plaques was observed. Biopsy of the plaques showed granulomatous infiltration of histiocytes. No definite diagnosis was made, and symptomatic relief occurred after a trial of empiric antimicrobial drug therapy. When the patient sought treatment again with persistent abdominal discomfort, endoscopy showed a lesion in the stomach that resembled a healed ulcer and numerous elevated yellow plaques throughout the colon and ileum. Microscopically, a granulomatous inflammation with macrophages filled with many acid-fast rods was seen, but mycobacterial growth did not occur in different media or in a guinea pig. Antituberculous treatment was initiated, and the patient slowly improved. A repeat colonoscopy showed fewer and smaller lesions. Efforts to culture the organism from biopsy specimens were again unsuccessful (different solid and liquid media, blood or chocolate agar, guinea pig, Balb/c mice). Sequencing of polymerase chain reaction (PCR) products of the 16S rRNA gene (*3*) from the organism represented a previously unknown mycobacterial species (EMBL DNA database: accession number Z50172). "*Mycobacterium tilburgii*" was proposed as the designation of this species because the novel mycobacterium was identified in the city of Tilburg (*4*). Retrospective analysis of the initial bladder biopsy specimen and of 2 lymph nodes taken during abdominal surgery (which became necessary because of a complicating ileal obstruction) confirmed the presence of "*M*. *tilburgii*" 16S rRNA gene sequences. All samples yielding PCR fragments hybridized with a "*M*. *tilburgii*"–specific biotinylated DNA probe.

A 34-year-old AIDS patient sought treatment for involuntary weight loss. Endoscopy showed white superficial embossments of the duodenal mucosa. Biopsy specimens were negative for acid-fast bacteria and mycobacterial growth (again, different solid and liquid media, extended incubation periods). Antiretroviral therapy was begun, but asthenia, persistent fever, diarrhea, vomiting, and cachexia developed. Repeat endoscopy showed yellow, plaquelike lesions in the duodenum (Figure A1) and esophagus with periodic acid–Schiff (PAS) and acid-fast intracellular bacteria that were nonculturable. PCR of 16S rRNA gene (*3*) confirmed the presence of mycobacterial DNA; sequencing showed 100% homology to the species with the proposed name "*M. tilburgii*" (*4*). Retrospectively, "*M*. *tilburgii*"16S rRNA was present in the initial duodenal biopsy specimen, when no mycobacteria were detected microscopically. Combination therapy led to a gradual disappearance of fever and diarrhea. The patient's weight increased, and the lesions disappeared (Table). Control endoscopy results were unremarkable, although duodenal biopsy specimens showed PAS-positive material. Mycobacterial DNA was no longer detectable.

**Table:** Characteristics of patients with confirmed "*Mycobacterium tilburgii*" disease*

Sex (age, y)	Immuno-suppression	CD4 count (cell/μL)	Disease manifestation	Treatment (duration, mo)	Diagnosed in	Reference
F (45)	No	Normal	Bladder, intestinal lymphnodes, stomach, ileum, colon	Pyrazinamide, isoniazid, rifampin (5); ethambutol, ciprofloxacin, clarithromycin, rifampin (11); surgical resection	The Netherlands	This study
M (34)	AIDS	37	Esophagus, duodenum	Ethambutol, clarithromycin, rifabutin (5)	Germany	This study
M (35)	AIDS	20	Duodenum, abdominal lymph nodes	Ethambutol, clarithromycin, rifabutin (12†)	Germany	(*9*)
M (41)	AIDS	17	Pulmonary nodules	Surgical resection; ethambutol, ciprofloxacin, clarithromycin (6); ethambutol, clarithromycin (12‡)	Germany	(*10*)

We believe that the identified acid-fast organism named "*M*. *tilburgii*" was the causative agent of illness in the 2 patients. First, the clinical and histopathologic appearance of the lesions is compatible with mycobacterial infection. Second, the finding that the macrophages in these lesions contained large numbers of acid-fast bacteria supports the conclusion that mycobacterial infection caused the lesion. Third, the involvement of normally sterile locations such as lymph nodes, and the improvement after antimycobacterial therapy with disappearance of the lesions, acid-fast organisms, and mycobacterial DNA supports a causative role of the organism rather than a bystander role.

Attempts to culture the organism from biopsy specimens and resection materials remained unsuccessful, although specific requirements for organisms like *M*. *genavense*, a mycobacterium that also is not cultivable on regular mycobacterial media (*5*), were met. Diagnosis therefore had to rely on sequencing of PCR products. Based on 16S rRNA homology, "*M*. *tilburgii*" shows close relationship to *M*. *sherrisii*, a *M*. *simiae*–related mycobacterium (*6*) that probably corresponds to *Mycobacterium* strain IWGMT 90143 (accession no. X88906) (*7*). "*M*. *tilburgii*" also has a high homology to *Mycobacterium* sp. Murphy, a mycobacterial species that is considered the cause of canine lepra and that also has not been cultivated so far (*8*) (accession no. AF144747).

These 2 patients double the number of patients reported previously (Table). Both previously reported *M. tilburgii* patients had AIDS, 1 of whom had a disease similar to that of the second patient in this report (*9*). Taken together, 3 patients had gastrointestinal involvement with yellow mucosal plaques (Figure A1), and 1 patient had pulmonary nodules (*10*). One of the 4 patients did not have a detectable immunodeficiency, yet had gastrointestinal and urinary bladder lesions. Although complications occurred, treatment was successful in all 4 cases with usual anti–*Mycobacterium avium* complex therapy. Interestingly, all 4 patients so far are of middle-European descent. This species may be geographically confined, similar to the occurrence of *M*. *malmoense* in which most clinical isolates come from northern Europe (*1*).

## Figures and Tables

**Figure A1:**
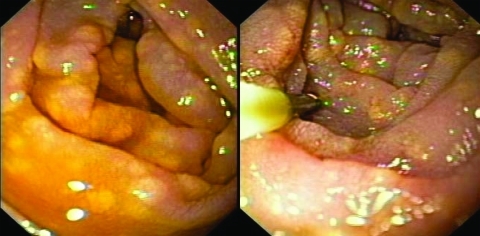
Yellow plaques in the mucosa of the duodenum in patient 2. Similar lesions had been observed in patient 1 in the colon, ileum, and bladder, whereas white lesion had been described in the other AIDS patient with gastrointestinal disease (*9*).
